# Basic beliefs about self and the world in people with suicidal ideation

**DOI:** 10.1192/j.eurpsy.2024.521

**Published:** 2024-08-27

**Authors:** T. I. Medvedeva, O. M. Boyko, O. U. Vorontsova, S. N. Enikolopov

**Affiliations:** ^1^Clinical psychology, Federal Stare Budgetary Scientific Institution “Mental Health Research Center”, Moscow, Russian Federation

## Abstract

**Introduction:**

Basic beliefs can be defined as a person’s implicit, global, stable ideas about the world and about himself. The psychological features of people with suicidal ideas can be considered as characteristics of the value sphere of a person who is ready to choose a destructive way of solving problems.

**Objectives:**

The relationship of basic beliefs regarding the general «Benevolence of the surrounding world», its «Meaningfulness» and «Worthiness of the Self» with the presence of suicidal ideas was investigated.

**Methods:**

The study involved 140 people, (117 women). The Janoff-Buhlman World Assumptions Scale (WAS), the short Epstein Rational-Experiential Inventory (REI)), Simptom Check List-90-Revised (SCL-90R), moral dilemmas (proposed by J.D.Green), as well as separate questions about the suicidal ideation, risk tendency were used.

For analysis, the subjects were divided into two subgroups: 98 people without thoughts of suicide; and 42 people answered that they had thoughts of suicide of varying severity. The subgroups did not differ by gender; in the group with suicidal ideation, the average age of the subjects was lower.

**Results:**

In the subgroup with suicidal ideas, almost all indicators of basic assumptions, such as “Benevolence of the World” (average values of 16.10 ± 3.28 and 12.13 ± 4.80 for the control subgroup and the subgroup with suicidal ideas), were lower: “Benevolence of the People “ (15.35±3.07 and 12.42±4.97), “justice” (12.46±3.30 and 10.46±3.60), “value of one’s own self” (16 .21±3.93 and 11.83±5.15), etc., with the exception of the “Self-controllability”, which does not differ between subgroups. Also, in the subgroup with suicidal ideas, the indicators of “randomness” were increased (15.67±3.64 and 18.67±3.96). Indicators on the clinical scale “Hostility” of the SCL-90R questionnaire are also significantly higher in the group with suicidal ideation (average values 0.53±0.5 and 1.29±0.8). In the group with suicidal ideation, there is a higher tendency to take risks. At the level of a statistical trend, the rational method of decision-making in the “Rational - Intuitive” questionnaire is lower (average values 14.3 and 13.0; significance level of differences 0.05). In the “Moral Dilemmas” test, in the subgroup with suicidal ideas, the ratio of choices in personal and impersonal dilemmas is statistically higher (0.67 and 0.93).

**Conclusions:**

It was shown that the presence of suicidal ideas is associated with a reduced indicator of the Worthiness of the Self, the meaningfulness of the world and its benevolence, and with an increased sense of randomness as a principle for distributing ongoing events, which can manifest itself in a propensity for risk, impulsive decisions, devaluation of human life.

**Disclosure of Interest:**

None Declared

